# Prevalence of Febrile Seizures in Children in Zahedan, South East of Iran

**Published:** 2019

**Authors:** Ghasem MIRI ALIABADI, Ali KHAJEH, Alireza OVEISI, Mahsa POORJANGI

**Affiliations:** 1 Department of Pediatrics, Children and Adolescent Health Research Center, Zahedan University of Medical Sciences, Zahedan, Iran

**Keywords:** Seizure, Fever, Prevalence, Children, Iran

## Abstract

**Objectives:**

Febrile seizure is the most common seizure disorder in childhood and a common cause of hospitalization in hospitals. We aimed to investigate the prevalence of febrile seizures in children in Zahedan, south-east of Iran.

**Materials & Methods:**

In this cross-sectional study, 600 children under 7 yr were examined for positive history of febrile seizure in 2014. The cluster sampling method was used, information was collected using a questionnaire, and data were expressed using descriptive- analytical tests.

**Results:**

The mean age of the children was 2.7±1.8 year. The sample consisted of 290 (48.3%) boys and 310 (51.7%) girls. Of the 600 children studied, 21 experienced febrile seizure and the incidence of febrile seizures was 3.5%. No significant difference was observed in terms of age or gender. Among the patients with history of febrile seizure, 2 (9.5%) had a positive family history of seizure. The age at the first febrile seizure was under one year in 13 patients (61.9%) and over one year in 8 patients (38.1%).

**Conclusion:**

Results indicated a moderate incidence of febrile seizure in the studied population. No significant difference was observed in terms of age or gender.

## Introduction

“Febrile seizures (FS) are the most common seizure in childhood” (1). FS occur in 2%-5% of children under age 5 year. However, the highest prevalence rate of 14% is reported (1). The peak incidence of FS is about 18 months of age. Previous family history of FS in siblings and parents is suggestive of a genetic predisposition (2). In 9%-35% of cases, FS is of the complex type (3). One-third of children with FS experience its relapse, while 10% of children experience three or more FS attacks (2, 4).

Intracranial infections and other definitive causes of seizures should be ruled out in FS (5, 6). In these patients, in most cases, fever is the result of upper respiratory system, gastroenteritis and urinary tract infections (1-3). On the other hand, there are cases of FS where seizure is observed before fever; therefore, factors other than fever must be involved in seizures.

Despite the relatively high incidence of this condition, it is a cause of concern to parents because of the concurrence of the two important phenomena of fever and seizure in children. Fortunately, despite what parents think, this disease is very benign and with good outcome in children and rarely causes brain injuries (7, 8).

The genetic basis of the disease and its mode of transmission are not exactly clear. However, most studies have pointed to its polygenic mode of inheritance. Nevertheless, the incidence of a particular HLA haplotype has not been observed in patients with FS (7, 9).

Diagnosis of this condition is essentially clinical and based on its description provided by parents. However, taking the detailed history of the patient including symptoms in the child, history of recent consumption of antibiotics or drugs given by the parents, and recent history of vaccination are helpful in determining the cause of fever, and examination for upper respiratory tract infections, which are the most common underlying cause of FS, must certainly be considered. However, the cause of fever in about one-third of these patients cannot be determined (10).

Studies on children with FS have reported significantly lower plasma ferritin levels (11). Plasma ferritin levels in these children were lower than in healthy children, but the difference was not statistically significant (12). This may suggest a possible role of iron deficiency in the pathogenesis of this condition. On the other hand, the prevalence of FS in children with thalassemia major who have high iron levels is very low (13).

No comprehensive study has been carried out so far on the prevalence of the disease in Zahedan; therefore, this study was designed to determine its prevalence in this city in 2014. 

## Materials & Methods

In this cross-sectional descriptive study, the statistical population consisted of the 1-month-old to 7-yr-old children in Zahedan, southwest Iran in 2014. 

Exclusion criteria included history of seizures without fever prior to FS, history of seizures caused by CNS infection, and lack of parental cooperation. 

The sample size was 600, and sampling was conducted through cluster sampling in Zahedan by visiting health centers. All data were collected through conducting face-to-face interviews with the parents and recording their responses in the questionnaires. 

Ethical principles including obtaining written consent from parents and the confidentiality of patient information were observed. 

The required data were entered into SPSS 16 (Chicago, IL, USA) and were analyzed using descriptive tests and the chi-square test.

## Results

Overall, 600 children aged 1 month to 7 yr were enrolled. The mean age was 33.2±22.1 months, and there were 290 (48.3%) boys and 310 (51.7%) girls. Of the 600 children studied, 21 patients (3.5%) had FS. Nine boys (3.1%) and 12 girls (3.9%) had FS, but no statistically significant difference was observed between the genders (*P*=0.388). The mean age of the children who had FS was 38.7±21.5 months, whereas the mean age of children without FS was 32.9±22.0 months (*P*=0.803).

Among patients with FS, 2 (9.5%) had a family history of seizures. Age at the first febrile seizure in 13 patients (61.9%) was less than one year of age, and in 8 patients (38.1%) was over one year of age. The most common symptoms mentioned by the patients’ parents were locking jaw (78%), tonic or clonic limb movements (84%), and upward gaze (88%).

## Discussion

In this study, the prevalence of FS was 3.5%. The mean age of children was 2.7 yr, and no significant difference was observed in terms of age and gender. Nine point five percent (9.5%) of the patients had a family history of seizures, and the first FS in most patients happened when they were less than one year of age. The incidence rate of febrile seizure in our study was consistent with those found in other studies where reported from 1.4%-4% (14-17). However, the reported incidence rates of FS are different in different parts of the world. For instance, it was 14% in Guam, 8.8% in Japan, 5%-10% in India (18), 0.35% in Hong Kong and 0.5%-1.5% in China (19). The peak age of the first FS was 2 yr (14). The mean age of children in the present study was higher than other studies (14,15, 17,); however, in our study, 60% of the FS had occurred under one year of age. In another study, most of the children (75.7%) had experienced the first seizure between the ages of 6 months to 3 year (15).

In this study, about 10% of the patients had a positive family history of seizures. In two similar studies, about 20% of children had positive family history of FS or epilepsy (14, 20). Different studies have reported positive family history of FS in 25%-40% of cases; however, in one study, 9.5% of children had a positive family history of FS (16,17, 21).

In our study, the ratio of girls to boys was not significantly different. This difference was not significant in other studies either (20, 22). Various studies have been conducted in Iran on the prevalence, etiology and geographical distribution of FS (20, 23-26). The inconsistencies in the results of various studies can be attributed to factors such as race, geographical differences, and diagnosis of FS and the method of selecting patients (27).

**In conclusion,** there was a moderate prevalence of FS in the studied population. No significant difference was observed in factors such as age and gender. Considering the results of this study, necessary training is provided to improve the knowledge and attitude of the parents and the caregiving teams, especially in relation to what to do when seizure attacks occur, to reduce seizure intensity in children and to mitigate stress and anxiety in parents. Studies with larger sample sizes and encompassing broader geographic regions should be carried out. 
